# Comprehensive genetic analysis of facioscapulohumeral muscular dystrophy by Nanopore long-read whole-genome sequencing

**DOI:** 10.1186/s12967-024-05259-8

**Published:** 2024-05-13

**Authors:** Mingtao Huang, Qinxin Zhang, Jiao Jiao, Jianquan Shi, Yiyun Xu, Cuiping Zhang, Ran Zhou, Wenwen Liu, Yixuan Liang, Hao Chen, Yan Wang, Zhengfeng Xu, Ping Hu

**Affiliations:** 1 Department of Prenatal Diagnosis, Women’s Hospital of Nanjing Medical University, Nanjing Women and Children’s Healthcare Hospital, 123 Tianfei Alley, Mochou Road, Nanjing, Jiangsu 210004 People’s Republic of China; 2 Department of Neurology, Nanjing First Hospital, Nanjing Medical University, Nanjing, Jiangsu 210006 People’s Republic of China

**Keywords:** Facioscapulohumeral muscular dystrophy, Long-read sequencing, Whole genome sequencing, Methylation, Single nucleotide variant

## Abstract

**Background:**

Facioscapulohumeral muscular dystrophy (FSHD) is a high-prevalence autosomal dominant neuromuscular disease characterized by significant clinical and genetic heterogeneity. Genetic diagnosis of FSHD remains a challenge because it cannot be detected by standard sequencing methods and requires a complex diagnosis workflow.

**Methods:**

We developed a comprehensive genetic FSHD detection method based on Oxford Nanopore Technologies (ONT) whole-genome sequencing. Using a case–control design, we applied this procedure to 29 samples and compared the results with those from optical genome mapping (OGM), bisulfite sequencing (BSS), and whole-exome sequencing (WES).

**Results:**

Using our ONT-based method, we identified 59 haplotypes (35 4qA and 24 4qB) among the 29 samples (including a mosaic sample), as well as the number of D4Z4 repeat units (RUs). The pathogenetic D4Z4 RU contraction identified by our ONT-based method showed 100% concordance with OGM results. The methylation levels of the most distal D4Z4 RU and the double homeobox 4 gene (*DUX4*) detected by ONT sequencing are highly consistent with the BSS results and showed excellent diagnostic efficiency. Additionally, our ONT-based method provided an independent methylation profile analysis of two permissive 4qA alleles, reflecting a more accurate scenario than traditional BSS. The ONT-based method detected 17 variations in three FSHD2-related genes from nine samples, showing 100% concordance with WES.

**Conclusions:**

Our ONT-based FSHD detection method is a comprehensive method for identifying pathogenetic D4Z4 RU contractions, methylation level alterations, allele-specific methylation of two 4qA haplotypes, and variations in FSHD2-related genes, which will all greatly improve genetic testing for FSHD.

**Supplementary Information:**

The online version contains supplementary material available at 10.1186/s12967-024-05259-8.

## Background

Facioscapulohumeral muscular dystrophy (FSHD) is an autosomal dominant neuromuscular disorder characterized by progressive and asymmetric weakening of facial, scapular girdle, and upper limb skeletal muscles [1, 2]. It is one of the most prevalent disorders of muscular dystrophy with a prevalence of 1:20,000 to 1:8,000 [3, 4]. FSHD has been categorized into two subtypes [5]. FSHD1, the predominant subtype, accounts for approximately 95% of cases and is attributed to an aberrant contraction in D4Z4 repeat units (RUs) in the 4q35 region [6, 7]. FSHD2 accounts for approximately 5% of cases and arises because of mutations in the epigenetic modifier genes *SMCHD1*, *DNMT3B*, or *LRIF1* [8]. The pathogenetic mechanism of FSHD has been attributed to aberrant expression of the double homeobox 4 gene (*DUX4*) in skeletal muscles resulting from aberrant hypomethylation status in the 4q35 region [9–11].

Genetic analysis for FSHD is challenging because of the long length and repetitive nature of the DNA sequence involved and the limited sequence differences between pathogenetic and non-pathogenetic alleles. In the general population, the 4q35 region contains 11–100 tandem copies of 3.3-kb CpG-rich D4Z4 RUs. The repeat contraction in FSHD1 reduces the number of repeats to between 1 and 10, resulting in epigenetic modification, chromatin relaxation, and increased expression of *DUX4*, which is partially encoded in the D4Z4 repeat [11]. A homologous sequence with 98% sequence identity to D4Z4 has also been identified in the 10q26 region, which presents a challenge for FSHD genetic diagnosis [12, 13]. Furthermore, the 4q35 region has two haplotypes, 4qA and 4qB, distal to D4Z4; however, only the 4qA allele contributes to stable expression of *DUX4* mRNA because of the presence of a polyadenylation signal in the most distal D4Z4 RU [2, 5].

Genetic diagnosis for FSHD1 has three requirements: (i) confirmation of the presence of a permissive haplotype, (ii) determination of the D4Z4 repeat length, and (iii) detection of the methylation status in patients without the D4Z4 repeat contraction. Southern blot is the traditional method used to detect D4Z4 repeat lengths and to differentiate the 4qA/4qB haplotypes [14]; however, it is a time-consuming procedure that is not suitable for large-scale clinical applications. Optical genome mapping (OGM) is a technique for FSHD1 detection [15, 16]. Because OGM can detect exceptionally long genomic variations, it offers superior detection of contractions in the D4Z4 repeat length for FSHD1. The third-generation single-molecule sequencing technology developed by Oxford Nanopore Technologies (ONT) is promising for diagnosing FSHD because of its long sequencing length and ability to simultaneously detect methylation [17–19]. Nanopore CRISPR/Cas9-targeted resequencing has also been applied to accurately measure the number of D4Z4 RUs and associated methylation status in patients with FSHD [20, 21]. However, for the FSHD2 subtype, DNA bisulfite sequencing (BSS) or next-generation sequencing is still needed for diagnosis. Therefore, comprehensive diagnosis of FSHD currently requires multiple technologies. This situation warrants the evaluation of new technologies with the potential to replace multiple technologies.

We developed a novel FSHD detection method based on ONT whole-genome sequencing for the comprehensive genetic analysis of FSHD that can distinguish the 4q35 and 10q26 D4Z4 repeat regions, determine the 4qA and 4qB haplotypes, identify pathogenetic contraction in D4Z4 RUs, detect the methylation status of the *DUX4* region, and call FSHD2-related gene mutations simultaneously. We applied ONT-based procedure to samples from 16 cases with FSHD1 and 13 healthy controls and compared the results with those from OGM, BSS, and whole-exome sequencing (WES). The results confirm that the comprehensive analysis of FSHD using our ONT-based method holds substantial promise in clinical application as a universal approach for diagnosing FSHD.

## Methods

### Subjects

Twelve clinically‑confirmed or suspected FSHD1 patients and 11 healthy adult controls from Nanjing Maternity and Child Health Care Hospital between December 2021 and March 2023 were respectively included in this study. Six human induced pluripotent stem cell (iPSC) lines (P2, P3, P6, P7, C2, and C4) generated from the peripheral blood of two clinically‑confirmed patients (P1 and P5) and two healthy adult controls (C1 and C3) were also included. The description of all samples is presented in Supplemental Table S1. All 29 samples were pregenotyped by OGM, and 27 of the samples were used for BSS because two of the samples did not have enough DNA for BSS. Nine of the samples were tested by WES and other samples did not have sufficient DNA. Written informed consent was obtained by a study-certified genetic counsellor before the samples were collected. The research Ethics Committee of Nanjing Maternity and Child Health Care Hospital approved the study.

### Nanopore whole-genome sequencing

Details of the Nanopore sequencing and base calling procedures have been described previously [22]. All 29 samples were sequenced using Nanopore PromethION devices with R9.4.1 flow cells (ONT, UK). We used the SQK-LSK109 kit (ONT, UK) and its recommended protocol to construct sequencing libraries, and 1 µg of input DNA per library and standard PromethION scripts for sequencing. At approximately 48 h, we performed a nuclease flush using the ONT recommended protocol, then reprimed the flow cell and added a fresh library for the same sample. Raw data were collected as FAST5 files and converted to FASTQ format using Guppy v5.0.16 (ONT, UK). Reads with quality scores < 9 and read lengths < 500 bp were filtered using NanoFilt v2.8.0 (https://github.com/wdecoster/nanofilt). The clean reads were aligned to the T2T CHM13v2.0 (https://github.com/marbl/CHM13) human reference genome using minimap2 v2.24 (https://github.com/lh3/minimap2). The median read length was 8.14 kb, and the median read quality was 14.2. The mean sequence depth for all samples was 29.3 × (Supplemental Table S1).

### Comprehensive analysis procedure of the ONT-based detection method

**Fig. 1:**
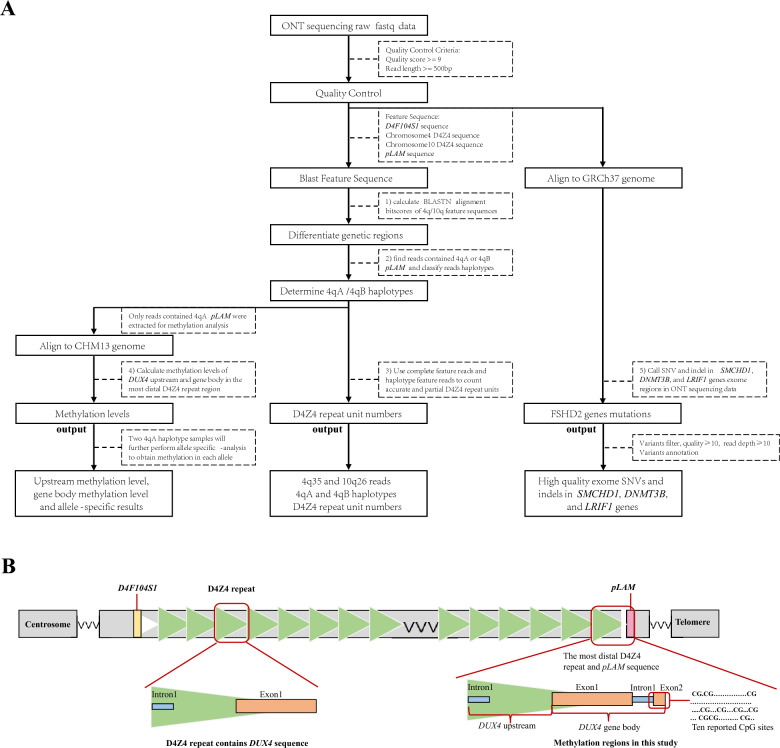
Workflow of ONT-based comprehensive genetic FSHD detection procedure. **A** Workflow of ONT-based FSHD detection method, from raw data processing to final output. **B** Schematic for 4q35 D4Z4 repeat region in T2T-CHM13 reference genome. The T2T-CHM13 reference genome’s repeat array region has 33 D4Z4 repeat units and the 4qA haplotype. Cartoon depicting the location of *D4F104S1* (yellow), the D4Z4 repeat array (green triangles), and *pLAM* (pink) from the 4qA haplotype sequence. The left inset panel shows a complete D4Z4 repeat unit, which contains an incomplete *DUX4* gene sequence (intron1 and exon1). The right inset panel shows the most distal D4Z4 unit and the complete *DUX4* gene structure. *DUX4* introns are indicated as blue squares, exons are indicated as orange squares. The *DUX4* upstream region is defined as the most distal D4Z4 unit to the *DUX4* gene body, and the complete *DUX4* gene is defined as the whole *DUX4* sequence. Ten reported CpG sites were shown in schematic diagram

Our novel ONT-based comprehensive analysis for FSHD detection procedure is shown in Fig. 1A. The procedure has five parts, (1) differentiating the 4q35 and 10q26 homologous regions, (2) determining 4qA and 4qB haplotypes, (3) identifying pathogenetic D4Z4 contractions, (4) calculating methylation level, and (5) calling FSHD2-related exome mutations.

(1) To differentiate the 4q35 and 10q26 homologous regions, BLASTN v2.11.0, (https://blast.ncbi.nlm.nih.gov/doc/blast-help/downloadblastdata.html) was used to align chromosome-specific feature sequences *D4F104S1*, D4Z4, and *pLAM* to each ONT reads. Then, the overall alignment bitscores of the 4q35 and 10q26 specific feature sequences in each read were calculated and the best overall alignment bitscores were used to classify reads as 4q35 or 10q26. *D4F104S1*, D4Z4, and *pLAM* sequences were downloaded from NCBI Nucleotide (https://www.ncbi.nlm.nih.gov/nuccore).

(2) To determine 4qA and 4qB haplotypes, the haplotype-specific *pLAM* sequence was aligned to each ONT read, which is similar to the procedure in part 1. Reads containing a 4qA *pLAM* sequence were classified as 4qA haplotype and reads containing a 4qB *pLAM* sequence were classified as 4qB haplotype.

(3) To identify pathogenetic D4Z4 contractions and precisely quantify the number of repeats, we defined complete feature reads as those that spanned from *D4F104S1* to *pLAM* and haplotype feature reads as those that contained only D4Z4 and *pLAM*. Complete feature reads were used to count accurate D4Z4 RU number, whereas haplotype feature reads can only partially quantify D4Z4 RU numbers. Reads contained *D4F104S1* and D4Z4 or only D4Z4, were defined as uncomplete feature reads. Those reads could use as non-pathogenetic markers, only if they contained ≥ 10 RUs. A schematic diagram of the 4q35 region is shown in Fig. 1B. The number of D4Z4 sequences aligned to each ONT read was counted to determine the D4Z4 RU number (**Supplementary Fig. S1**).

(4) To calculate methylation levels, we performed read-level methylation base calling on ONT FAST5 files. We used the T2T CHM13v2.0 genome as the reference and called the methylation level of each CpG site using Megalodon v2.5.0 (https://github.com/nanoporetech/megalodon) with Guppy v5.0.16 (ONT, UK) with the Rerio (https://github.com/nanoporetech/rerio) modified base model. The most distal D4Z4 repeat region in the T2T CHM13v2.0 genome was extracted as the methylation targeted region. A schematic diagram of the methylation region is shown in Fig. 1B. The average methylation levels of *DUX4* upstream and gene body were calculated as:

Single-read plots were generated from modbamtools (v0.4.8, https://rrazaghi.github.io/modbamtools). Blue point represents unmethylated CpGs and red point represents 5-methyl CpGs.

(5) To call FSHD2-related exome variants, ONT reads were aligned to the reference genome sequence (GRCh37/hg19) by minimap2. DeepVariant (PEPPER-Margin-DeepVariant r0.8, https://github.com/kishwarshafin/pepper) was used to call exome single nucleotide variants (SNVs) and small indels (< 50 bp) in three FSHD2 related genes, *SMCHD1*, *DNMT3B*, and *LRIF1*. The same exome targeted region file from WES testing was used to keep variants in gene’s exome and flanking regions. Other variants out of exome targeted region were not included in this analysis. Quality control was performed to filter variants with quality values < 10 and read depths < 10 in the DeepVariant output. The population frequency filter was not set in this analysis.

### Optical genome mapping

For each individual, high molecular weight genomic DNA was isolated from fresh blood samples collected in EDTA tubes or iPSCs using a Bionano Prep™ Blood and Cell Culture DNA Isolation Kit (Bionano Genomics, USA). Ultra-high molecular weight DNA was fluorescently labeled with DLE-1 enzyme (Bionano Genomics, USA) using a DLS DNA Labelling Kit (Bionano Genomics, USA). Labeled DNA was loaded onto a Saphyr® chip (to collect 1300 Gb of molecules > 150 kb) and imaged on a Saphyr® instrument. Data were processed with Bionano Solve software v3.5 to align labeled molecules against the reference sequence predicted label pattern; the hg38 human reference genome carries both the 4qA and 4qB D4Z4 haplotypes. Molecules that aligned to the reference 4q35 or 10q26 region were collected to generate representative allelic profiles of structural variation and used to interpret FSHD genotypes by the custom EnFocus FSHD analysis v1.0 (Bionano Genomics, USA). Samples with insufficient data were further analyzed by de novo assembly for full genomes. Selected regions of the genome were assembled and analyzed as part of the quality control process.

### DNA methylation analysis

For the bisulfite reaction, 1000 ng of genomic DNA was converted using a EZ DNA Methlyation-Lightning Kit (Zymo Research, USA) following the manufacturer’s instructions. Then 2 µL of converted products were amplified using Q5U Hot Start High-Fidelity DNA Polymerase (NEB, USA) according to the manufacturer’s instructions. PAS-specific PCR was performed in a total volume of 50 µL as follows: 30 s at 98 °C, 35 × (10 s at 98 °C, 30 s at 65 °C, 30 s at 72 °C), and 2 min at 72 °C. The 4qA-allele-specific primers were from Calandra et al. [23]. The obtained PCR products were purified using a FastPure Gel DNA Extraction Mini Kit (Vazyme, China). Purified PCR products were cloned into a pCE2 TA/Blunt-Zero vector using a 5 min TA/Blunt-Zero Cloning Kit (Vazyme, China) and transformed into *Escherichia coli* DH5α Electro-Cells.

At least 50 clones were chosen at random from each sample, and individual clones were sent for Sanger sequencing (Tsingke Biological Technology, China). Ten previously reported CpG sites were included as methylation markers. The methylation level for each site was calculated as ratio of methylated sites to total sites. The mean methylation level for the 10 CpG sites was calculated as the average level across the 10 sites. A schematic diagram of the 10 CpG sites is shown in Fig. 1B.

### Exome sequencing analysis

Genomic DNA was extracted from nine of the samples for exome sequencing. Libraries were prepared using an Agilent SureSelect XT Library Prep Kit (Agilent, USA) and exon capture was performed using Agilent SureSelect XT Human All Exon v6 (Agilent, USA). The captured DNA was amplified by PCR and paired‐end sequenced on an Illumina HiSeq 2500 platform (2 × 150 bp read length) (Illumina, USA). Sequencing reads were aligned to the reference genome sequence (GRCh37) by BWA v0.7 (https://github.com/lh3/bwa). The Broad Institute’s GATK v4.0 (https://gatk.broadinstitute.org) and VEP v108 (https://useast.ensembl.org/info/docs/tools/vep/index.html) were used to call SNVs and small indels (< 50 bp) and to annotate the variants. Quality control was performed to filter variants with quality values < 20 and read depths < 20 in the GATK output. The population frequency filter was not set. Exome variants in *SMCHD1*, *DNMT3B*, and *LRIF1* were extracted using intersectBed (bedtools v2.30.0, https://bedtools.readthedocs.io/en/latest/) for subsequent analysis.

### Statistical analyses

Two-sided *P* values < 0.05 were considered statistically significant. Correlation analyses were performed by Pearson’s correlation test. Two group comparisons were performed by t-test. Area under the ROC Curve (AUC) was calculated by receiver operating characteristic (ROC) curve analysis. All analyses were performed using R software v4.2.1 (The R Foundation for Statistical Computing, http://www.cran.r-project.org).

## Results

### Analysis of 4q haplotypes and D4Z4 RU numbers

Given that only contractions of D4Z4 RUs in 4q35 are related to the development of FSHD, we first differentiated homologous genomic regions of 4q35 and 10q26. Based on chromosome-specific feature sequences BLAST results, we compared chromosome-specific Bitscores of each ONT reads and successfully categorized the ONT reads into the 4q35 and 10q26 regions (Supplemental Table S2). Similarly, using a haplotype-specific feature sequence, 35 permissive 4qA and 24 non-permissive 4qB haplotypes were detected in the 4q35 region from 16 cases and 13 controls (Table 1). Using the paired OGM test, we found that the permissive and non-permissive haplotypes were 100% consistent with the ONT results (Supplemental Table S3).

**Table 1:** Summary of D4Z4 RU and haplotype results of 4q35 derived from ONT and OGM

ID	Chr	ONT		OGM		Pathogenetic (repeats ≤ 10) accordance
		Repeat Units	Haplotype	Repeat Units	Haplotype	
P1	chr4	2	4qA	2	4qA	Y
		≥ 19	4qB	27	4qB	Y
P2	chr4	2	4qA	2	4qA	Y
		≥ 12	4qB	28	4qB	Y
P3	chr4	2	4qA	2	4qA	Y
		≥ 14	4qB	28	4qB	Y
P4 (mosaic)	chr4	2	4qA	2	4qA	Y
		≥ 31	4qA	37	4qA	Y
		18	4qB	18	4qB	Y
P5	chr4	4	4qA	4	4qA	Y
		12	4qA	12	4qA	Y
P6	chr4	4	4qA	4	4qA	Y
		≥ 11	4qA	12	4qA	Y
P7	chr4	4	4qA	4	4qA	Y
		12	4qA	12	4qA	Y
P8	chr4	6	4qA	6	4qA	Y
		≥ 13	4qA	29	4qA	Y
P9	chr4	6	4qA	6	4qA	Y
		≥ 15	4qB	21	4qB	Y
P10	chr4	7	4qA	7	4qA	Y
		≥ 11	4qA	26	4qA	Y
P11	chr4	7	4qA	7	4qA	Y
		7	4qB	7	4qB	Y
P12	chr4	8	4qA	8	4qA	Y
		≥ 16	4qB	17	4qB	Y
P13	chr4	8	4qA	8	4qA	Y
		≥ 20	4qB	24	4qB	Y
P14	chr4	9	4qA	9	4qA	Y
		15	4qB	15	4qB	Y
P15	chr4	9	4qA	9	4qA	Y
		≥ 15	4qB	24	4qB	Y
P16	chr4	9	4qA	9	4qA	Y
		≥ 20	4qA	24	4qA	Y
C1	chr4	12	4qA	12	4qA	Y
		≥ 13	4qB	17	4qB	Y
C2	chr4	≥ 10	4qA	12	4qA	Y
		17	4qB	17	4qB	Y
C3	chr4	14	4qA	14	4qA	Y
		≥ 14	4qA	17	4qA	Y
C4	chr4	14	4qA	14	4qA	Y
		18	4qA	17	4qA	Y
C5	chr4	≥ 11	4qA	17	4qA	Y
		≥ 21	4qB	18	4qB	Y
C6	chr4	21	4qA	21	4qA	Y
		18	4qB	18	4qB	Y
C7	chr4	≥ 11	4qA	24	4qA	Y
		≥ 12	4qB	27	4qB	Y
C8	chr4	≥ 16	4qA	28	4qA	Y
		≥ 14	4qB	14	4qB	Y
C9	chr4	≥ 18	4qA	33	4qA	Y
		≥ 12	4qB	32	4qB	Y
C10	chr4	≥ 27	4qA	34	4qA	Y
		≥ 17	4qB	26	4qB	Y
C11	chr4	13	4qB	13	4qB	Y
		≥ 13	4qB	29	4qB	Y
C12	chr4	≥ 15	4qB	16	4qB	Y
		≥ 15	4qB	16	4qB	Y
C13	chr4	20	4qB	20	4qB	Y
		≥ 18	4qB	22	unknown^a^	Y

a unknow means OGM FSHD analysis is unable to determine the haplotype

To further ascertain pathogeneticity, we calculated the numbers of D4Z4 RUs in the 4q35 region. In the ONT analysis, complete feature reads that spanned from *D4F104S1* to *pLAM* accurately counted D4Z4 RUs. Haplotype feature reads achieved only partial quantification of the D4Z4 RU. We identified 30 alleles that contained complete feature reads (4qA:23, 4qB:7) and 29 alleles that contained haplotype feature reads (4qA:12, 4qB:17). For accurate numbers, we identified 2–21 RUs. The longest RU for partial quantification was ≥ 31. We successfully separated the D4Z4 RUs into pathogenetic or non-pathogenetic allele groups using 10 RUs as the threshold. More importantly, using our ONT-based method, we were able to obtain the accurate number of D4Z4 RUs in pathogenetic alleles in all 16 cases, showing 2–9 RUs in the 4qA allele (Table 1). There was 100% concordance between the ONT and OGM results (Supplemental Table S3). Although limited by read length, we still obtained accurate numbers for 58.33% of non-pathogenetic 4qA alleles and 29.17% of non-permissive 4qB alleles using ONT sequencing. Conversely, OGM detected the accurate numbers of all the D4Z4 RUs in all the 4qA and 4qB alleles (Table 1).

Mosaicism is common in FSHD, and, in this study, we identified a mosaic family. Our ONT results showed that this family has a mosaic father (P4) who has two RUs in the pathogenetic 4qA allele and ≥ 31 RUs in the non-pathogenetic 4qA allele, and 28 RUs in the 4qB allele. The mother (C7) is a healthy control with 24 RUs in the 4qA allele and 27 RUs in the 4qB allele. The proband (P1) inherited the two RUs in the pathogenetic 4qA allele from the father (Table 1**, **Fig. 4C). OGM then confirmed the accurate number of RUs of the father’s non-pathogenetic 4qA allele to be 37.

In addition to the 4q35 region, we analyzed haplotypes and D4Z4 RU numbers in the 10q26 region. All the alleles had 10qA haplotypes in the ONT and OGM tests. As was done for the 4q35 region, our ONT-based method counted accurate numbers of alleles with RUs ≤ 10 and correctly distinguished whether alleles contain > 10 RUs (Supplemental Table S4).

### Analysis of average methylation levels in the *DUX4* upstream and gene body regions

**Fig. 2:**
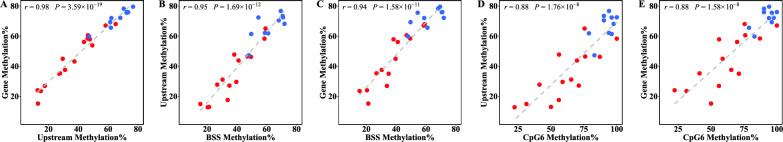
Correlation between average methylation levels in the *DUX4* upstream region, gene body, BSS (mean values of 10 CpG sites) and CpG6. **A** Scatter plot of average methylation levels in the *DUX4* upstream region and gene body. **B** Scatter plot of average methylation levels in the *DUX4* upstream region and BSS. **C** Scatter plot of average methylation levels in the *DUX4* gene body and BSS. **D** Scatter plot of average methylation levels in the *DUX4* upstream region and CpG6. **E** Scatter plot of average methylation levels in the *DUX4* gene body and CpG6. Samples from cases and controls are shown as red and blue dots, respectively

To assess the capability of our ONT-based method to detect the epigenetic status of FSHD, we calculated the average methylation levels in the *DUX4* upstream and gene body regions for all permissive 4qA haplotype alleles and compared them with the BSS results for 10 CpG methylation sites (Table 2). The average methylation levels in the *DUX4* upstream region and the gene body are significantly correlated (r = 0.98, *P* = 3.59 × 10^−19^) (Fig. 2A). Importantly, they are both highly correlated with the mean methylation value of the 10 CpG sites (upstream: r = 0.95, *P* = 1.69 × 10^−12^, gene body: r = 0.94, *P* = 1.58 × 10^−11^) (Fig. 2B, C) as well as with the GpG6 site, which is considered the most informative CpG site (upstream: r = 0.88, *P* = 1.76 × 10^−8^, gene body: r = 0.88, *P* = 1.58 × 10^−8^) (Fig. 2D, E).

**Table 2:** DNA methylation in case and control samples by ONT and BSS

ID	Upstream^a^	Gene body^b^	BSS^c^	Site1	Site2	Site3	Site4	Site5	Site6	Site7	Site8	Site9	Site10	D4Z4 repeat units in 4qA haplotype allele
P1	12.75	24.02	20.45	14.93	34.33	11.94	11.94	14.93	22.39	25.37	20.90	20.90	26.87	2
P2	17.56	26.92	33.80	20.00	48.00	12.00	40.00	20.00	56.00	62.00	32.00	26.00	22.00	2
P3	12.95	15.19	21.35	9.62	38.46	0.00	15.38	11.54	50.00	40.38	21.15	15.38	11.54	2
P4	65.03	67.99	59.18	44.90	67.35	38.78	75.51	44.90	75.51	63.27	71.43	48.98	65.31	2/37 (mosaic)
P5	27.76	35.27	26.83	11.11	33.33	7.94	20.63	23.81	41.27	39.69	33.33	23.81	33.33	4
P6	14.90	23.51	15.49	7.84	39.22	1.96	17.65	5.88	31.37	19.61	13.73	13.73	3.92	4
P7	31.11	37.57	30.38	11.54	48.08	5.77	38.46	9.62	65.38	46.15	26.92	38.46	13.46	4
P8	49.38	53.85	–	–	–	–	–	–	–	–	–	–	–	6
P9	47.73	57.86	38.25	24.56	71.93	10.53	36.84	24.56	56.14	45.61	45.61	38.60	28.07	6
P10	46.23	58.40	49.43	22.86	54.29	24.29	54.29	35.71	87.14	71.43	55.71	34.29	54.29	7
P11	27.13	35.02	35.00	19.23	34.62	0.00	26.92	9.62	71.15	73.08	51.92	44.23	19.23	7
P12	37.42	43.16	–	–	–	–	–	–	–	–	–	–	–	8
P13	43.79	56.12	41.09	28.30	60.38	16.98	33.96	32.08	69.81	54.72	0.00	28.30	45.28	8
P14	46.45	60.55	47.00	20.00	52.00	16.00	36.00	40.00	76.00	80.00	46.00	54.00	50.00	9
P15	29.56	44.94	39.61	25.49	64.71	5.88	27.45	35.29	58.82	72.55	54.90	25.49	25.49	9
P16	58.30	67.04	58.46	25.49	84.31	33.33	70.59	62.75	100.00	54.90	66.67	35.29	62.75	9
C1	47.25	59.72	48.01	16.98	73.58	20.75	56.60	45.28	83.02	73.58	0.00	24.53	37.74	12
C2	73.42	75.86	70.59	74.51	76.47	49.02	80.39	52.94	90.20	84.31	0.00	62.75	64.71	12
C3	62.14	71.98	58.80	14.00	82.00	24.00	72.00	54.00	94.00	78.00	56.00	48.00	66.00	14
C4	61.87	65.58	60.96	50.00	71.15	44.23	69.23	53.85	78.85	78.85	59.62	53.85	50.00	14
C5	61.39	69.25	49.62	13.46	40.38	11.54	59.62	67.31	96.15	69.23	40.38	46.15	51.92	17
C6	72.36	75.51	54.42	17.31	53.85	21.15	51.92	40.38	96.15	67.31	61.54	63.46	71.15	21
C7	70.44	78.23	68.04	70.59	78.43	13.73	68.63	68.63	90.20	90.20	76.47	74.51	49.02	24
C8	76.69	79.54	69.41	39.22	88.24	56.86	66.67	54.90	94.12	76.47	76.47	64.71	76.47	28
C9	72.48	75.97	70.94	41.51	86.79	32.08	79.25	71.70	100.00	84.91	71.70	62.26	79.25	33
C10	68.15	72.11	71.90	40.48	80.95	47.62	85.71	59.52	97.62	88.10	80.95	66.67	71.43	34
C11	–	–	–	–	–	–	–	–	–	–	–	–	–	Not 4qA
C12	–	–	–	–	–	–	–	–	–	–	–	–	–	Not 4qA
C13	–	–	–	–	–	–	–	–	–	–	–	–	–	Not 4qA

^a^Upstream is defined as the region from the most distal D4Z4 unit to *DUX4* gene body, average methylation levels calculated by ONT results

^b^Gene body is defined as the region of the complete *DUX4* gene, average methylation levels calculated by ONT results

^c^BSS shows the average methylation sequence results of 10 representative CpG sites using sodium bisulfite sequencing, following 10 sites are representative CpG sites using in this study

**Fig. 3:**
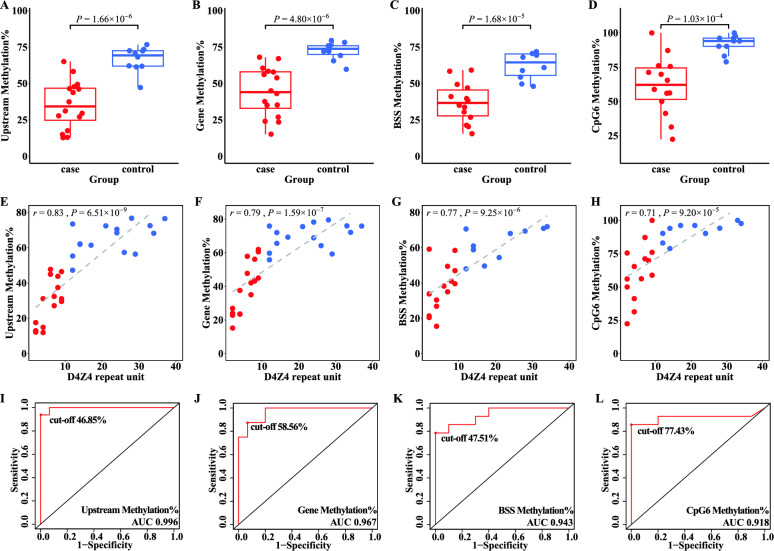
Methylation levels in distinguishing cases and controls. **A**–**D** Box plots show the difference in average methylation levels of the *DUX4* upstream region (**A**), gene body (**B**), BSS (mean values of 10 CpG sites) (**C**), and CpG6 (**D**) between cases (red) and controls (blue). Scatter plots show correlations between average methylation levels and D4Z4 repeats unit numbers. **E**–**H** The *DUX4* upstream region (**E**), gene body (**F**), BSS (**G**), and CpG6 (**H**) are shown in the plots. Each point represents a 4qA allele in the scatter plots of the upstream region and gene body plot. Each point represents a sample in the scatter plots of BSS and CpG6. Samples from cases and controls are shown in red and blue, respectively. **I-L** ROC curve analysis of the *DUX4* upstream region (**I**), gene body (**J**), BSS (**K**), and CpG6 (**L**) methylation levels are illustrated

We then focused on whether the average methylation levels in the *DUX4* upstream region and the gene body could distinguish cases and controls. The average methylation levels were 35.50% in cases and 66.62% in controls in the *DUX4* upstream region, and 44.21% in cases and 72.37% in controls in the *DUX4* gene body. The differences in average methylation levels between cases and controls are significant (Fig. 3A, B). The BSS methylation mean value of 10 CpG sites and the value of the CpG6 site show similar results (Fig. 3C, D). Correlation analysis shows significant correlations between the average methylation levels and RUs (upstream: r = 0.83, *P* = 6.51 × 10^−9^, gene body: r = 0.79, *P* = 1.59 × 10^−7^) (Fig. 3E, F). Similar significant correlations are found between the mean methylation value of 10 CpG sites and RU numbers (r = 0.77, *P* = 9.25 × 10^−6^), and between CpG6 and RU numbers (r = 0.71, *P* = 9.20 × 10^−5^) (Fig. 3G, H). Notably, the average methylation levels of the *DUX4* upstream region and the gene body show even higher correlation with the mean methylation value of the 10 CpG sites and CpG6, as indicated by the r values. These results strongly confirm the important role of methylation status in FSHD.

To further assess the potential use of methylation status for FSHD diagnosis, we performed a ROC curve analysis by comparing methylation levels in the 16 cases and 13 controls. The analysis shows the average methylation level of the *DUX4* upstream region detected pathogenetic alleles with a sensitivity of 1 and a specificity of 0.938 at the cut-off of 46.85% (AUC = 0.996) (Fig. 3I). The average methylation level of the *DUX4* gene body detected pathogenetic alleles with a sensitivity of 0.933 and a specificity of 0.875 at the cut-off of 58.56% (AUC = 0.967) (Fig. 3J). The BSS methylation results of 10 CpG sites (AUC = 0.943) and CpG6 (AUC = 0.918) also distinguish cases from controls (Fig. 3K, L); however, the AUC values are lower than those for the ONT methylation results. Our methylation markers have excellent diagnostic efficiency for cases and controls.

### Allele-specific methylation analysis of 4qA haplotype

**Fig. 4:**
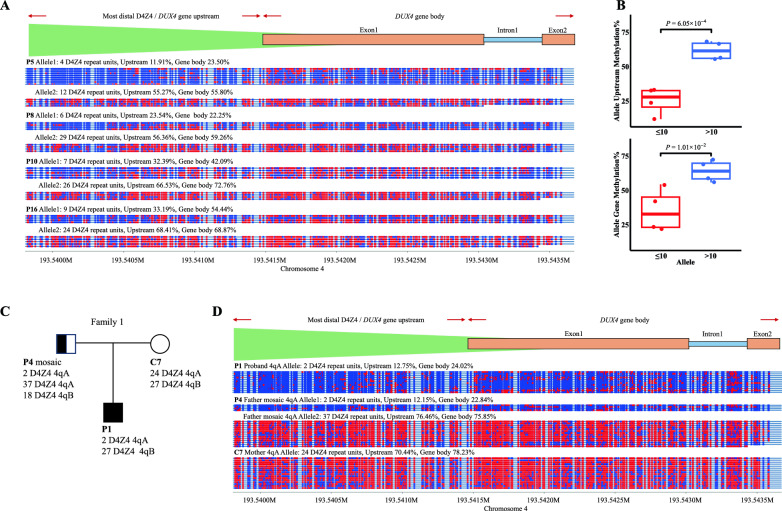
Allele-specific methylation analysis of 4qA haplotype. **A** Methylation plot of four cases with two 4qA haplotype. Single-read plots were generated from modbamtools (https://rrazaghi.github.io/modbamtools/). Blue points represent unmethylated CpGs and red points represent 5-methyl CpGs. **B** Box plots show the difference between the average methylation levels of the *DUX4* upstream region and the gene body within the range of ≤ 10 (red) and > 10 (blue) alleles. **C** Pedigree of family P1. **D** Methylation plot of 4qA 2, 37, 24 D4Z4 repeat units reads from Family 1. Family 1 possesses a D4Z4 repeat contraction and methylation plot of samples in the most distal D4Z4 repeat unit and *DUX4* gene. The father is a mosaic sample and has two pathogenetic D4Z4 repeats with a 4qA haplotype. The mother has two non-pathogenetic alleles, and the proband inherits the two paternal pathogenetic D4Z4 repeats

Classical BBS calculates the average overall methylation level of two alleles, which can lead to an overestimation of methylation when pathogenetic and non-pathogenetic 4qA alleles are present. The ONT-based method can detect the haplotype, D4Z4 RU number, and methylation status for each sequenced read, which not only allows the overall methylation level to be computed but also enables precise methylation assessment at the read level. In this study, four of our samples had pathogenetic and non-pathogenetic 4qA alleles, and the ONT overall methylation level of the 4qA alleles in the *DUX4* upstream region were 27.76%, 49.38%, 46.23%, and 58.30% in the four samples (Table 2). Using a cut-off value of 46.85% (calculated above), pathogenetic 4qA alleles would not have been identified in two of the samples only based on methylation levels. We then conducted an allele-specific methylation analysis to precisely identify the methylation status of samples with two 4qA haplotypes. In these four samples, we found that methylation levels of the pathogenetic 4qA alleles (4, 6, 7, and 9 RUs) in the *DUX4* upstream region were 11.91%, 23.54%, 32.39%, and 33.19%, whereas in the non-pathogenetic 4qA alleles (12, 29, 26, and 24 RUs) the values were 55.27%, 56.36%, 66.53%, and 68.41% (Fig. 4A). These differences between the methylation status of the pathogenetic and non-pathogenetic alleles in the four samples are also significant (upstream, *P* = 6.05 × 10^–4^) (Fig. 4B). The methylation status is consistent with D4Z4 RUs. Moreover, using the same cut-off value of 46.85%, all the alleles were correctly classified as pathogenetic or non-pathogenetic. Methylation levels in *DUX4* gene body gave the same results (Fig. 4A, B).

Allele-specific analysis is especially important in mosaic samples. In the mosaic family (Fig. 4C), the mosaic sample P4 had two RUs in pathogenetic 4qA and 37 RUs in non-pathogenetic 4qA. BSS results were unable to distinguish between the two 4qA alleles (overall methylation values of 10 CpG: 59.18%, CpG6: 75.51%) (Table 2), showing that the pathogenetic allele present in the mosaic sample was also obscured. The ONT results gave the overall methylation levels for both 4qA alleles as 65.03% (upstream) and 67.99% (gene body) (Table 2), also indicating that hypomethylation of the pathogenetic allele was obscured by the methylation level of the non-pathogenetic allele. Conversely, the allele-specific methylation analysis results for the 4qA haplotype showed that the average methylation levels of the two RUs in pathogenetic 4qA allele were 12.15% (upstream) and 22.84% (gene body), and those of the 37 RUs in non-pathogenetic 4qA allele were 76.46% (upstream) and 75.85% (gene body) (Fig. 4D). These results demonstrate a distinct difference in methylation levels between pathogenetic and non-pathogenetic 4qA alleles in the P4 sample.

### Analysis of exome variants of FSHD2-related genes

To fully leverage the advantages of ONT whole-genome sequencing, we analyzed variations in the exon regions of three reported pathogenetic genes (*SMCHD1*, *DNMT3B*, and *LRIF1*) associated with FSHD2 and compared the results with those from WES. Nine samples were included for analysis by the ONT-based method and WES. Based on the same exome targeted regions from WES testing, ONT identified 17 SNVs (Supplemental Table S5); 15 were common variants and two were rare variants, LRIF1 c.1233 T > G, and DNMT3B c.1297 + 6G > A. WES also identified the same 17 SNVs. The ONT-based method detected 100% of the SNVs identified by WES in each sample (Supplemental Table S6).

## Discussion

Comprehensive genetic characterization of FSHD using conventional methods is challenging. We developed an ONT-based method to achieve a genetic–epigenetic integrated analysis of FSHD and evaluated its performance using a case–control study design with 16 cases and 13 control samples. We show that this method effectively differentiates homologous regions, haplotypes, pathogenetic D4Z4 RU contractions, methylation alterations, and genetic mutations, with high consistency and additional advantages compared with conventional OGM, BSS, and WES methods.

One of the challenges of molecular genetic analysis for FSHD1 is identifying the D4Z4 RU contractions of a permissive 4qA haplotype in the 4q35 region. We show that the molecular characteristics of FSHD1 alleles identified by our ONT-based method closely match those identified using OGM. Diagnosed 4qA-derived contracted reads (≤ 10 RUs) were found in all cases, whereas no such diagnosed contracted reads were found in the controls. In addition to the contracted reads, we occasionally obtained reads with > 10 replicates from non-pathogenetic alleles. The results of our ONT-based method are consistent with those of previous studies on the diagnosis of FSHD using Nanopore sequencing [24]. We expected read lengths to be adequate for detecting pathogenetic D4Z4 repeat contractions. And read lengths did prove to be accurate enough while using our ONT-based method to simultaneously detect the size of 4q-derived D4Z4 RUs and for haplotyping 4qA/4qB.

Mosaicism is common in FSHD and has been found in 14%–20% of unaffected parents of patients with de novo FSHD [25–28]. In addition to mosaicism in parents, a high frequency (26%) of somatic mosaicism has been found in patients with de novo FSHD [28]. Detailed analysis of somatic and germline mosaicism carrier states in families with de novo FSHD is required to achieve accurate genetic counseling. Southern blot analysis can identify some mosaicisms but may miss low-level mosaicisms. Stence et al. [16] reported that OGM identifies a higher rate of somatic mosaicism (5.1%) than the 1.5% rate detected by Southern blot. One of the patients in our study had a pathogenetic allele that was inherited from their asymptomatic low-level mosaic father. The ONT-based method successfully captured four complete feature reads from low abundance pathogenetic alleles. Although ONT-based method cannot determine mosaicism ratios, its unique advantage is its ability to capture low abundance mosaic alleles with contracted D4Z4 RUs.

Methylation status can predict penetrance, disease severity, and rate of progression of FSHD [23, 29–31].The CpG methylation status of the D4Z4 sequence, especially the most distal D4Z4 RU, serves as a reliable marker for FSHD diagnosis. Traditional methylation assays use 4qA allele-specific FasPAS primers for BSS, analyzing 10 CpG sites in the most distal D4Z4 RU [23]. CpG6 is considered the most informative site because it can distinguish cases and evaluate phenotypes [23, 29–31]. In our analysis, we used ONT-based sequencing methylation data to calculate average methylation levels of the *DUX4* upstream region and gene body in the most distal D4Z4 RU, showing high correlation with 10 CpG sites and provided better results than BSS in the three following aspects: First, the ONT-based method provided average methylation levels of the *DUX4* upstream and gene body that had higher correlation with RUs and better AUCs in distinguishing affected samples compared with BSS. Second, the ONT-based method allows for simultaneous acquisition of genomic and methylation data with no extra costs compared with BSS. Finally, BSS is a time-consuming and laboratory-intensive technology, whereas ONT methylation assay needs only bioinformatic analysis.

Another advantage is that ONT-based methylation data can be used to perform allele-specific methylation analysis in samples with two 4qA haplotypes. Previous studies have shown up to 40% of 4qA alleles in the population, implying that this is high prevalent [32, 33]. The possession of two 4qA haplotypes is common, thus hypomethylation of the pathogenetic allele may be overshadowed by hypermethylation of the non-pathogenetic allele, resulting in an inconclusive methylation status outcome. Classical BSS cannot separate pathogenetic from non-pathogenetic 4qA haplotypes. We show that the high methylation level of the non-pathogenetic allele can obscure the pathogenetic allele in our mosaic sample. For example, in the mosaic sample, CpG6, the most informative CpG site, the methylation level was 75.51%, which is considered a non-pathogenetic methylation value. Using our ONT-based method, the allele-specific methylation analysis found an average methylation level of 12.15% for the two D4Z4 RU alleles and 76.46% for the 37 D4Z4 RU alleles in the *DUX4* upstream region. Thus, the separately computed methylation levels show greater precision than the overall methylation level computed for the two 4qA alleles.

Nanopore CRISPR/Cas9-targeted resequencing has been proposed for diagnosing FSHD [20, 21]. Targeted sequencing of chromosome 4q/10q regions with high sequencing depths is a cost-effective method. Our ONT-based whole-genome sequencing procedure provides a comprehensive view of the entire genome, and therefore, in addition to genetic testing for FSHD, our method can potentially be used to simultaneously detect other known muscular dystrophies [34]. For example, although samples from patients with FSHD2 were not included in this study, our results show that our ONT-based method can accurately detects FSHD2-related genes’ mutations. Moreover, Nanopore CRISPR/Cas9-targeted resequencing requires complex experimental procedures and can only be conducted in select laboratories. Conversely, ONT-based whole-genome sequencing is technically simpler and does not require specialized bioinformatics tools and expertise in CRISPR/Cas9 technology.

Nonetheless, our study has certain limitations. Most importantly, ONT-based whole-genome sequencing generates limited valid reads for the 4q35 region, which hinders the determination of accurate RU numbers in healthy controls. Second, FSHD2 samples were not included. A large cohort study of patients with FSHD is needed to more fully explore the advantages of our ONT-based method. Third, the current cost of Nanopore third-generation sequencing remains relatively high.

## Conclusions

In conclusion, our study offers a novel and comprehensive strategy for FSHD diagnosis using ONT-based whole-genome sequencing. We have shown that our ONT-based method can achieve precise genotyping of 4q haplotypes, identify pathogenetic D4Z4 contractions, and detect methylation alterations and sequence variations in FSHD2-related genes in one step. Compared with the traditional approaches, our ONT-based method provides a more comprehensive, accurate, and efficient approach for FSHD genotyping. With the rapid development of the ONT techniques, ONT-based detection holds promise as a crucial tool for FSHD diagnostics in the near future.

### Supplementary Information

Supplementary Material 1: Fig. S1. Workflow for the procedure of identifying pathogenetic D4Z4 contractions and precisely quantifying the number of repeats.

Supplementary Material 2.

## Data Availability

All relevant data generated during this study will be made available by the corresponding author upon reasonable request.

## Abbreviations

FSHD: Facioscapulohumeral muscular dystrophy
ONT: Oxford nanopore technologies
OGM: Optical genome mapping
BSS: Bisulfite sequencing
WES: Whole-exome sequencing
RU: Repeat unit
iPSC: Human induced pluripotent stem cell
SNV: Single nucleotide variant
ROC: Receiver operating characteristic curve
AUC: Area under the ROC curve
*DUX4*: Double homeobox 4
*SMCHD1*: Structural maintenance of chromosomes flexible hinge domain containing 1
*DNMT3B*: DNA methyltransferase 3 beta
*LRIF1*: Ligand dependent nuclear receptor interacting factor 1
