# Supplementary figures and images for: Comprehensive genetic analysis of facioscapulohumeral muscular dystrophy by Nanopore long-read whole-genome sequencing
Source: J Transl Med. 2024 May 13;22:451. doi: 10.1186/s12967-024-05259-8 (PMC11092085; doi:10.1186/s12967-024-05259-8)

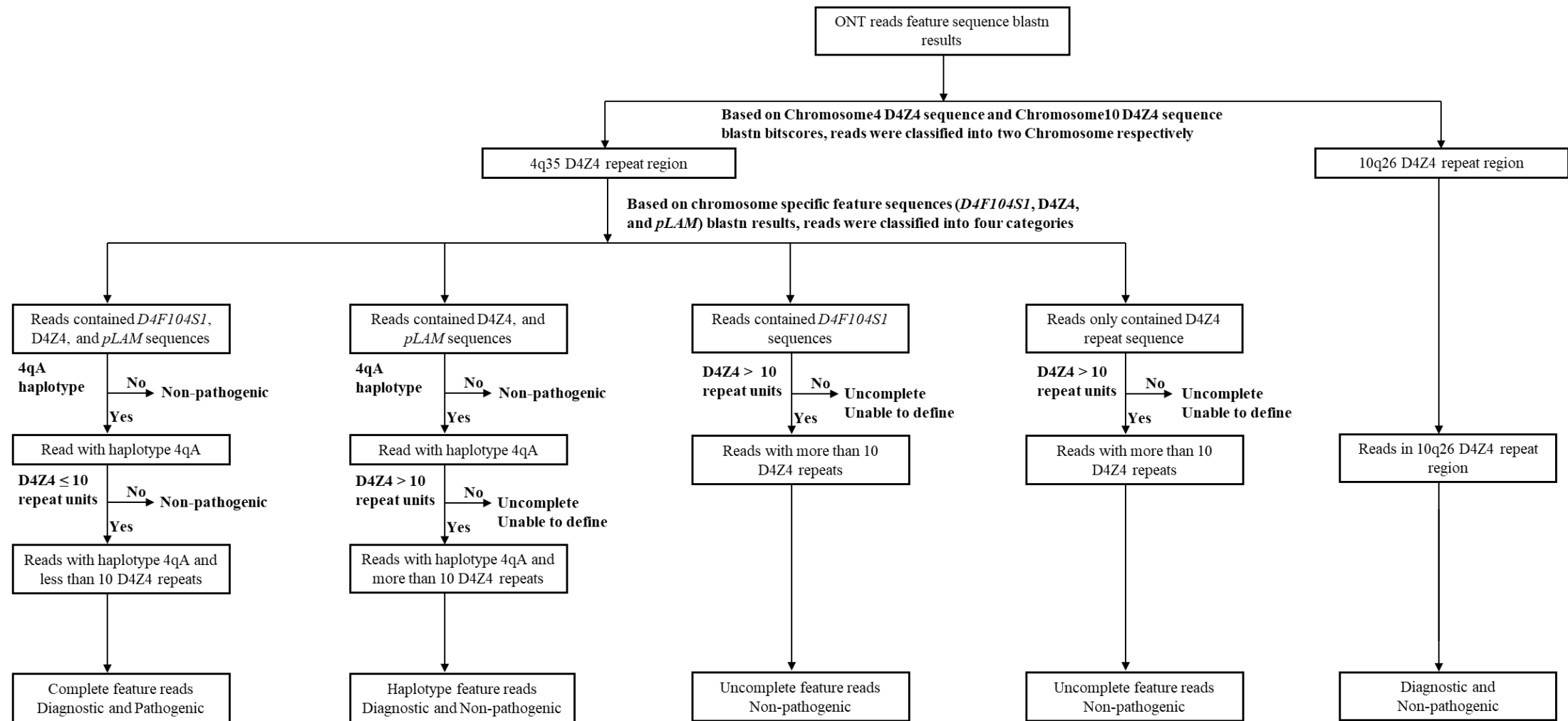

Supplement: Supplementary file 1 — Supplementary Material 1: Fig. S1. Workflow for the procedure of identifying pathogenetic D4Z4 contractions and precisely quantifying the number of repeats. [file 12967_2024_5259_MOESM1_ESM.pdf]
